# Assessment of the Effects of Economic Sanctions on Iranians’ Right to Health by Using Human Rights Impact Assessment Tool: A Systematic Review

**DOI:** 10.15171/ijhpm.2017.147

**Published:** 2018-01-20

**Authors:** Fatemeh Kokabisaghi

**Affiliations:** ^1^Healthcare and Law Department, School of Health Policy and Management, Erasmus University Rotterdam, Rotterdam, The Netherlands.; ^2^Department of Management Sciences and Health Economics, School of Health, Mashhad University of Medical Science, Mashhad, Iran.

**Keywords:** Economic Sanctions, Right to Medicine, Human Rights, Iran, HRIA Tool

## Abstract

**Background:** Over the years, economic sanctions have contributed to violation of right to health in target countries. Iran has been under comprehensive unilateral economic sanctions by groups of countries (not United Nations [UN]) in recent years. They have been intensified from 2012 because of international community’s uncertainty about peaceful purpose of Iran’s nuclear program and inadequacy of trust-building actions of this country. This review aimed to identify the humanitarian effects of the sanctions on the right of Iranians to health and the obligations of Iran and international community about it.

**Methods:** To assess economic sanction policies and identify violated rights and the obligations of states according to international human rights laws, in this study, Human Rights Impact Assessments (HRIA) tool is used. Applying this tool requires collection of evidences regarding the situation of rights. To provide such evidence, a systematic review of literature which involved 55 papers retrieved from the web-based databases and official webpages of Iran’s government and UN’ health and human rights committees and organizations was done. All articles about the consequences of economic sanctions related to nuclear activities of Iran on welfare and health of Iranians published from January 2012 till February 2017 in English and Persian languages were included. Search terms were economic sanctions, embargoes, Iran, welfare, health and medicine. Additional studies were identified by cross checking the reference lists of accessed articles. All selected papers were abstracted and entered into a matrix describing study design and findings, and categorized into a framework of themes reflecting the areas covered (health and its determinants). According to HRIA framework, related obligations of Iran and other states about adverse effects of the sanctions on Iranians’ right to health were extracted.

**Results:** The sanctions on Iran caused a fall of country’s revenues, devaluation of national currency, and increase of inflation and unemployment. These all resulted in deterioration of people’s overall welfare and lowering their ability to access the necessities of a standard life such as nutritious food, healthcare and medicine. Also, the sanctions on banking, financial system and shipment led to scarcity of quality lifesaving medicines. The impacts of sanctions were more immense on the lives of the poor, patients, women and children. Humanitarian exemptions did not protect Iranians from the adverse effects of sanctions.

**Conclusion:** Countries which imposed economic sanctions against Iran have violated Iranians’ right to health. International community should have predicted any probable humanitarian effects of sanctions and used any necessary means to prevent it. Furthermore, Iran should have used any essential means to protect people from the adverse effects of sanctions. Now, they should work on alleviation of the negative effects of sanctions. Even though, some of the effects such as disability and death cannot be compensated. In future, before imposition of sanctions, decisions makers should advice an international order to prevent such impacts on targeted countries’ populations.

## Introduction

Since the World War I, sanctions have often been applied by international organizations and nations as a routine policy tool to react to any nation’s actions that they oppose.^1^ Economic sanctions seem to be more humane ways of resolving international disputes than wars. However, multiple studies on Iraq, the former Yugoslavia, Nicaragua, Burundi, Cuba, and Haiti showed that due to their long term impacts on the lives and health of a large population, the adverse humanitarian effects of economic sanctions are comparable to, if not more immense than wars.^2-7^ Through exacerbating economic situation and functions of social systems of a target country, they decrease the access of people to necessities of life such as nutritious food and medical care.^3-5,7^ From practical point of view, there is no difference between dying due to being shot or being deprived from life-saving medicines. Islamic Republic of Iran (hereafter; Iran) has been under economic sanctions for more than three decades. In this study, the effects of the sanctions on Iranians’ right to health, as well as international human rights obligations of Iran and international community regarding this issue are scrutinized.

## Right to Health

According to the UN Declaration of Human Rights 1948, everyone has a right to a standard of living adequate for his health and well-being including food, medical care and social security without any kinds of discrimination on any grounds such as gender, race and the political, jurisdictional or international status of the place to which a person belongs.^8^ Right to health has been reflected in several international human rights treaties such as International Covenant on Economic social and Cultural Rights (ICESCR) Article 12. This right is a right to “the highest attainable standard of physical and mental health” (hereafter; right to health) based on ICESCR.^9^ Achievement of this level of health is one of the most important universal social goals. In the Constitution of World Heath Organization (WHO), health of all human beings is defined as a necessary condition to the attainment of universal peace.^10^

According to ICESCR, the right to health includes a right to access timely and appropriate healthcare and underlying determinants of health, such as safe water, nutritious food, housing, and healthy environment. All the facilities, services and goods related to health and its determinants should be of good quality, acceptable, available and physically and financially accessible to all, without any kinds of discrimination. States are needed to provide health insurance and financial aids for the poor to enjoy this right.^11^ The Committee on Economic, Social and Cultural Rights (ESCR) acknowledged resources limitations of states in realizing ESCRs in a limited time. Therefore, it required the states to realize minimum core obligations immediately after ratifying the covenant and progressively realize these rights by taking steps to pursue the goals of the Covenant and using the maximum of available resources.^9^ The core obligations of the right to health includes access to health facilities, products and services and minimum essential food, basic shelter, sanitation and safe water.^12^ While recognizing the right of the states (who lack recourses for provision of the minimum of rights) to international assistance, the Committee requires all the states parties to realize right to health and contribute to the improvement of international health.^9^

## Economic Sanctions and Human Rights

Sanctions are “measures taken by a state to coerce another to conform to an international agreement or norms of conduct, typically in the form of restrictions on trade.”^13^ These measures are called countermeasures which are resorted against an international wrongdoer in the case that they are not decided by UN Security Council.^14^ They may be comprehensive which prohibit commercial activities entirely with a country, or targeted (or smart) which block transactions of and with certain businesses, groups, or individuals of a target country.^15^ According to the Articles 39-43 of the Charter of UN 1945, if the Security Council determines any threat to the peace, breach of the peace, or act of aggression, it can decide what measures shall be taken to maintain or restore international peace and security. These measures may include use of arm forces, complete or partial interruption of economic relations and of rail, sea, air, postal, telegraphic, radio, and other means of communication, and the severance of diplomatic relations. All members of the UN are required to collaborate on these issues with the Council.^16^

In 2003, because the International Atomic Energy Agency (IAEA) was uncertain about the scope and nature of Iran’s nuclear activities, it asked Iran to be transparent and build confidence and suspend all enrichment related and reprocessing activities including research and development. In 2006, IAEA declared that it is “unable to make progress in its efforts to provide assurances about the absence of undeclared nuclear material and activities in Iran.”^17^ Therefore, the case of Iran was brought to UN Security Council. At first, Iran was needed to “build the confidence regarding to peaceful purpose of its nuclear program and to suspend all enrichment-related and reprocessing activities, including research and development.” However, Iran’s trust building attempts were not adequate according to the UN Security Council. In 2007, Iran confronted with the Council’s sanction resolution related to its nuclear activities.^18^ All the sanctions defined by Security Council against Iran have been about limitation on its nuclear and military industry. No economic sanctions against this country were initiated by the Council.^19^ However, some countries decided to use “coercive diplomacy” and unilaterally boycott Iran with economic sanctions in 2012. About the measures are to be taken by the member states of UN in order to maintain international peace and security, the Charter of UN clearly states that “the measures shall be concluded between the Security Council and Members or between the Security Council and groups of Members and shall be subject to ratification by the signatory states in accordance with their respective constitutional processes.”^16^ General comment no. 8 of ICESCRs about The relationship between economic sanctions and respect for economic, social and cultural rights indicates that:

Whatever the circumstances, such sanctions should always take full account of the provisions of the International Covenant on Economic, Social and Cultural Rights. The Committee does not in any way call into question the necessity for the imposition of sanctions in appropriate cases in accordance with Chapter VII of the Charter of the UN or other applicable international law. But those provisions of the Charter that relate to human rights (Articles 1, 55 and 56) must still be considered to be fully applicable in such cases.^20^

Sanctions are called “brutal instruments” by the UN Food Program; WHO has asked international community to ban them altogether.^21^ The Committee on the Rights of the Child also declared that economic sanctions can act as an obstacle to the implementation of the Convention on the Rights of the Child (CRC).^22^ Through humanitarian exemptions for food and medicine, the sanctions often do not aim to violate people’s right to health in target countries. But, still civilians of target countries suffer from deprivation; since, it is not possible to separate effects of economic sanctions on health and economy. UN Human Rights Council in 2013 declared that there are reliable evidences about serious consequences of sanctions on the rights of people particularly vulnerable groups such as women, children, the elderly, the poor, minorities, indigenous people and persons living with disabilities.^23^

Economic sanctions on countries such as Iraq, the former Yugoslavia, Nicaragua, Burundi, Cuba, and Haiti were associated with the deterioration of health and welfare of the population.^3,4,7^ In sanctions period, decline of revenues, increase of poverty, unemployment, and inflation, as well as deterioration of health services’ functions, school attainment and society’s development were reported. Also, the sanctions have made essential goods more costly and difficult to produce and maintain.^3^ In these situations, people’s ability to afford health services and maintain a healthy life style has been reduced.^5^ Rise of maternal, infant and child mortality rates has been considerable during sanctions period in some countries under sanctions. In addition, poor nutrition and lack of access to health services and medical supplies brought severe public health problems such as epidemics of diseases particularly among the poorest groups of the society.^24^ Furthermore, shortage of medicines and medical equipment deteriorated the practices of health systems. For instance, in Syria, sanctions brought difficulty in the import of essential medicines which were not produced locally.^25^ Cuba also lost access to raw materials needed for manufacturing pharmaceuticals and lacked the currency to purchase medicines and medical equipment from international market in sanctions period.^6^ Moreover, sanctions on import of non-medical products and spare parts, and trade restriction on water and electrical supply systems affected effectiveness of health systems in Cuba, Iraq, and Haiti; trade embargoes on agricultural sector such as fertilizers and seeds caused food shortage.^5^ In another case, reduction of revenues of target countries has decreased governments’ ability to finance healthcare system or sanctions on opening LC (letter of credit) for Iranian banks and shipment of imported goods caused shortage of medicines in Iran. Therefore, to ensure access of people to food and healthcare, humanitarian exemptions and providing supplementary aid are not adequate.

## Sanctions Against Iran

Poverty alleviation and social and health equity are prioritized in the Constitution and development plans of Iran. After the Revolution 1979, a welfare state system which focuses on health, education and social aid has been established in Iran. As results of a vast system of subsidies, material poverty has fallen significantly in this country. By improving urban infrastructures such as providing electricity, safe water and sanitation and universal free education, Iran has improved the living conditions of Iranians to a great extent. In 2011, more than 95% of Iranians had access to improved drinking water sources and sanitation facilities. Total adult literacy rate was 85% in this year.^26^ Also, for many years, Iran’s government provided subsidized essential food stuffs such as flour, rice, cooking oil, sugar and milk to all the population. In 2010, this country changed this policy to cash payment to everyone. Moreover, through establishing successful primary healthcare network around the country, health outcomes of the country have improved notably over recent decades. Life expectancy of Iranians increased from 63 to 73.3 during 1990-2012 and the rates of maternal, prenatal and child mortality have fallen considerably. Maternal mortality per 100 000 live birth decreased from 91 to 24.6 and infant mortality per 1000 live birth decreased from 44 to 15 in this period. Communicable diseases are controlled and are no longer the cause of mortality. Together, they cause less than 5% of deaths.^26,27^ UN Children Found (UNICEF) declared in 2011 that through a strong health and education network and infrastructure, Iran is on track to achieve most of the Millennium Development Goals’ targets including addressing poverty and hunger, primary education, child mortality and maternal health.^28^ However, regarding the reduction of poverty the country is facing major challenges such as increase of people in need of support because of conditions including inflation and unemployment.^27^

In recent decades, people of Iran, having an oil-dependent economy and inefficient industry continuously faced numerous challenges including the effects of Revolution 1979, eight-year-war with Iraq and several kinds of international sanctions from agriculture to the airline Industry. After the Revolution, the sanctions were mainly imposed by the United States. However,their effects were limited, since Iran could find ways to compensate for the loss partly through other countries or by some mediators despite these involving higher expenses. Sanctions imposed by UN Security Council which aimed at forceing Iran to stop its nuclear activates targeted the military and nuclear industry of Iran. However, without mandate of the UN, the United States, the European Union (EU) and some other countries decided to impose comprehensive multilateral restrictions on any cooperation with Iran in foreign trade. Embargos from the United States also included “secondary sanctions” on countries and companies doing business with Iran.^29,30^

**Figure 1:**
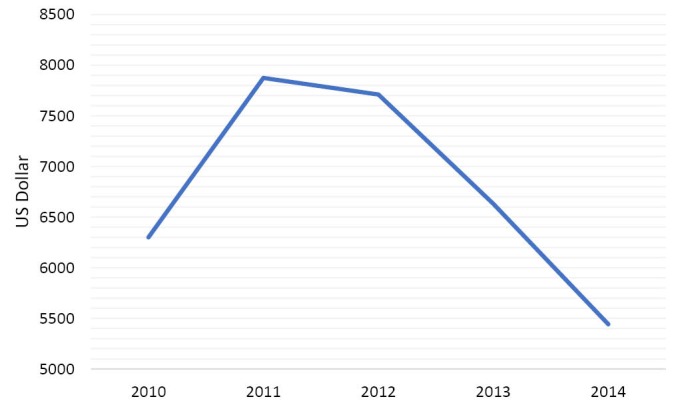
Iran’s Gross Domestic Product Per Capita (US Dollar) During 2010-2014.
Refernce: World Bank, available at: http://data.worldbank.org/country/iran-islamic-rep?view=chart.

**Figure 2:**
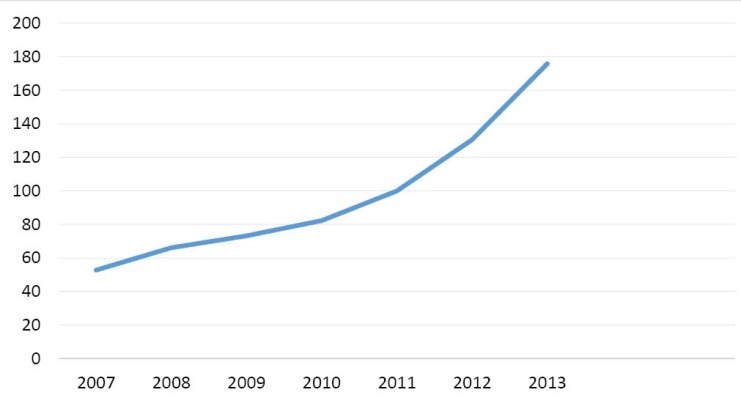
Consumer Price Index 2007-2013 Iran.
Reference: Trading Economics, available at: https://tradingeconomics.com/iran/consumer-price-index-cpi/forecast.

**Figure 3:**
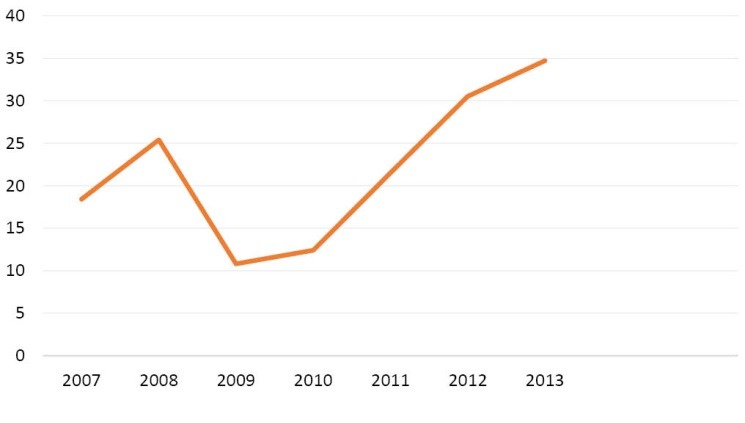
Infaltion Ratio 2007-2013 Iran.
Reference: Trading Economics, available at: https://tradingeconomics.com/iran/consumer-price-index-cpi/forecast.

**Figure 4:**
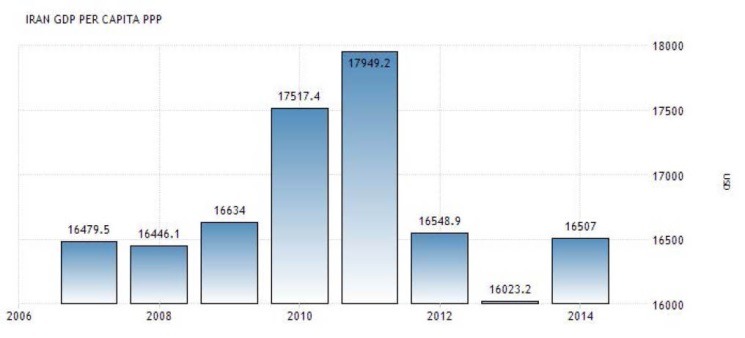
Iran’s GDP Per Capita PPP 2007-2014.
For about a decade, PPP has been influenced by several factors such as change of government policies on subsidized public services as well as economic sanctions which resulted in reduction of oil revenues, devaluation of national currency, decrease of export revenues, limitations on import of raw materials necessary for industry, bankruptcy of industry, unemployment and inflation. Significant decrease of GDP in 2013 and 2014 is related to refusal of main purchasers of Iranian oil to deal with Iran. In the last years of the sanctions period, Iran could find countries which were willing to buy Iran’s oil and could repair GDP.

When sanctions (imposed without the mandate of UN) were intensified in 2012 to target all sectors of Iran’s economy, the country’s ability to sell oil became limited. As an oil-dependent country, Iran’s revenues and financial ability to purchase needed supplies in the world market decreased considerably. It became worse after the freezing of properties of Iran’s Central Bank and other financial institutions in third countries. Sharp declines in oil revenues and industrial production, severe restrictions on the import of items, shipment and payment channels, and considerable devaluation of the national currency (the Riyal), caused high rate of inflation in every sectors of Iran’s economy.^31^ Also, Iran had to accept payment in gold, local currencies and bartered goods from a few Asian countries that still bought Iran’s oil. Therefore, Iran’s access to the US dollar and the euro needed for import from most of countries became limited. Furthermore, sanctions cut off Iranian banks from global financial system; international banks which dealt with Iran faced severe restrictions by international community.^32^ It made the transferring of oil’s earning back to the country extremely difficult. As a result, Iran had to process the transactions by intermediary banks that was very difficult and expensive.^31^ These all diminished Iran’s industry and economy and deteriorated Iranians’ welfare to a great degree. Gross domestic product (GDP) per capita decreased by 35% during 2012 -2014 (Figure 1).^33^ The consumer price index increased from 100 to 178 (Figure 2) and the inflation rate from 20 to 38% during 2011-2013 (Figure 3). GDP per capita purchasing power parity (PPP) decreased by more than 10% from 2011 till 2013 (Figure 4).^34^ Minimum wage decreased from US$275.4 in 2010 to US$155 in 2012 (Table 1).^35^ While the unemployment rate was 11.3 in 2016. This indicator was 10.5 in 2008.^36^

Reference: Trading Economics, available at: https://tradingeconomics.com/iran/gdp-per-capita-ppp.

Abbreviations: GDP, gross domestic product; PPP, purchasing power parity.

**Table 1:** Minimum Wage 2005-2014 Iran

**Year**	**Minimum Wage ($)**	Minimum Wage Based on Free Market Rate* ($)	**Annual Raise Compared to the Previous Year (%)**	** Inflation Rate of Year Before (%)**
2005	135.6	135.6	14	15.2
2006	162.6	162.2	18	10.4
2007	195.7	195.7	22	11.9
2008	227.3	227.3	17	18.4
2009	263.5	263.5	18	25.4
2010	275.4	275.4	13	10.8
2011	173.8	173.8	9	12.4
2012	155.8	111.3	18	21.5
2013	194.8	192.3	25	30.5
2014	243.5	202.9	25	36.7

Reference: Al-monitor available at: http://www.al-monitor.com/pulse/originals/2014/03/iran-wages-inflation-economy-law-protest.html.

In this article, after introducing the methods of study for assessing the humanitarian effects of sanctions and identifying materials that form the basis of the analysis, the adverse effects of economic sanctions on Iran’s economy, living conditions of Iranians and situation of right to health and medicine are reviewed. Next, the actions and legal obligations of Iran’s government and of the international community in the process of sanctions’ management are analyzed. Finally, some recommendations for future sanction regimes, in order to better respond to humanitarian effects of the sanctions are provided.

## Methods

In this study, to assess the adverse effects of economic sanctions on people’s right to health and identify the related national and international obligations related to this violation, Human Rights Impact Assessments (HRIA) tool is used. To tackle with the adverse impacts of trade agreements on the right to health, policy-makers employed various impact assessment tools such as Sustainability Impact Assessment of EU Trade Agreements. However, traditionally these tools focus on economic and environmental and not social effects. HRIA is preferred because it uses a legally binding framework of International human rights law which is based on a strong normative consensus and universally agreed principles. Also, it evaluates a full range of internationally agreed human rights, while focuses on empowerment and improvement. HRIAs tools emerged in the late 1990s for anticipating and measuring impacts of policies and programs on different human rights. They are helpful in identifying various types of duty- and right-bearers and their responsibilities. HRIAs have been applied in a broad range of different fields such as in development (by the Norwegian Agency for Development Cooperation and Food and Agriculture Organization [FAO]), Health (by UN Special Rapporteur of Right to Health), trade (by UN Bodies and national Parliaments) and multinational cooperations (by UN Global Compact, UN Human Rights Council).^37^

**Figure 5:**
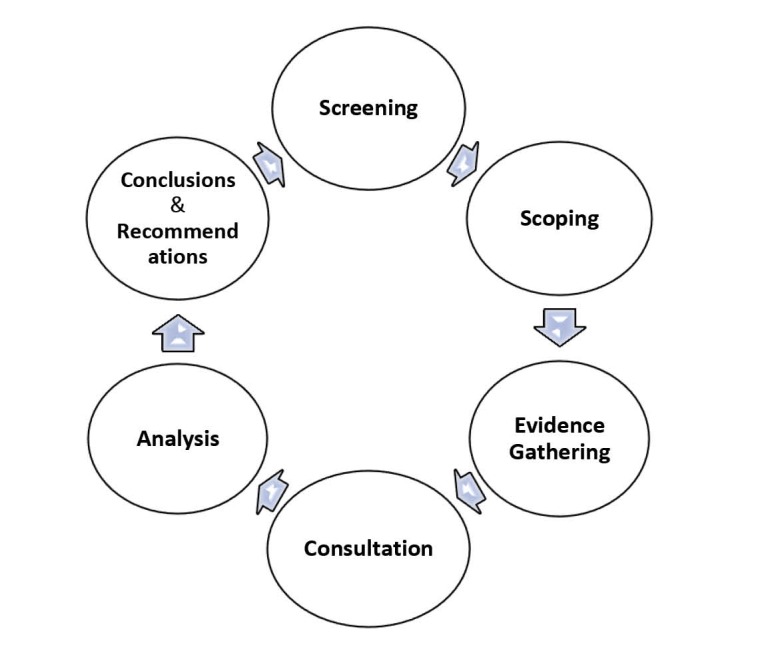
Human Rights Impact Assessments (HRIA) Tool.^37^

HRIAs are based on legal framework of human rights and promote accountability which is one of the key contributions of a human rights perspective.^38^ The purpose of this tool is to identify any inconsistency between international human rights obligations and other national and international obligations. HRIA identifies human rights violations which can be challenged by judicial bodies and ignoring them might cause significant legal consequences for violating states and institutions. In this assessment, human rights obligations are extracted from main human rights laws such as Universal Declaration of Human Rights 1948, International Covenant on Economic, Social and Cultural Rights (ICESCR) 1966, General Comment no. 14 of ICESCR on right to health and General Comment no. 8 ICESCR on the relationship between sanctions and right to health. HRIA includes 8 steps for policy-makers and 6 steps (as below) for scholars:

- Screening: It requires selecting key human rights issues that are most likely to be affected. In this study, the screening is done through analyzing the effects of economic sanctions on Iran and other countries.
- 1) Scoping: in this step, identifying the information needed and formulating concrete questions are necessary.
- 2) Evidence Gathering: A quantitative as well as qualitative research techniques can be applied in this step. In this study, content analysis of relevant papers has been done.
- 3) Consultation: interviewing affected populations and other potential right-holders or using secondary material, such as reports, studies and experiences which provide primary data is necessary. In this study, a literature review about the effects of the sanction policy on Iranians is applied.
- 4) Analysis: in this step, deciding over the concrete human rights impact of the policy assessed by analyzing the results of the literature review should be done. In the case that sanctions affected availability, accessibility, acceptability and quality of health (and its determinants) services, products and facilities, violation of the right to health has been occurred.
- 5) Conclusions and Recommendations: in this step, identifying specific duty-bearers and assigning them concrete responsibilities should be done (Figure 5).

### Search Strategy

The question of this study is that what the implications of economic sanction policies of 2012 against Iran on Iranians’ right to health are. A qualitative case study design involving structured document review of relevant articles and policy documents were undertaken. Two sets of literature were studied; the first set aimed to obtain papers focusing on the situation of Iranians’ enjoyment of their right to health; while the second set aimed to understand legal human rights obligations of Iran and countries involved in implementing sanctions about Iranian’s right to health and a standard of life.

### Selection Criteria

In order to define keywords for search in databases, several articles about the subject were analyzed. Collected documents included original articles, reviews, editorials, letters to the editor, interviews, short reports and communications. The papers that described the effects of economic sanctions on Iranian’s right to health published between January 2012 and February 2017 were included. They were written in English and in the Persian languages. Articles related to sanction’s effects on other countries, sanctions which were not about Iran’s nuclear program and non-economic sanctions were excluded. The papers related to justification of sanctions against Iran were included in the study if they discuss the health effects of sanctions. Moreover, papers about sanctions relating to human right violations of the Iranian government were not considered.

### Data Extraction

**Figure 6:**
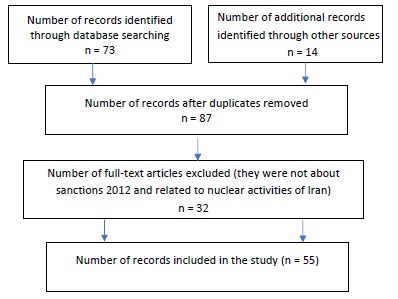
PRISMA Flow Diagram.

Data about the situation of Iranian’s enjoyment of their right to health in sanction period were collected from web-based databases including EBESCO, PubMed, Web of Science, Scopus, Emerald, Elsevier, Cochrane library, Hein online, J Store, Project Muse, Science Direct Springer, Wiley Online Library, Oxford Journals, Embase, SID, and Google Scholar by searching key words: “economic sanctions,” “right to health,” “embargoes,” “medicine,” and “Iran.” More data was found by cross checking the reference lists of accessed articles. Furthermore, official webpages of Iran’s government and UN’ health and human rights committees and organizations were studied to find the results of economic sanctions against Iran on people’s right to health. Selection of papers was exhaustive to locate every available one about the subject of the study. The collected papers were analyzed in-depth in order to collect evidences of humanitarian impacts of economic sanctions on Iranians’ lives and right to health. A total of 87 documents including papers (n = 76), books (n = 5), and reports (n = 6) on the humanitarian effects of economic sanctions were identified. The abstracts were reviewed and duplicated articles, or those that were not pertinent to the study (because they were not about effects of sanctions on health and its determinants) or did not adequately address the impacts of sanctions on Iranian’s livelihood (meaning that they did not clarify how the right to health is influenced) were put aside. 55 documents emerged to be related to the topic (Figure 6). The other part of study is about the obligations of targeted and targeting states about right to health. For this part, electronic databases including UN Treaty Collections and UN official Document System were searched following the terms “human rights,” “right to health,” “embargoes,” “medicine,” and “economic sanctions.” The number of relevant international laws that were identified was 13. All selected documents were summarized and categorized in two main parts; effects of sanctions on Iranian’s right to health and the obligations of Iran and international community about protection of Iranians’ right to health.

### Data Analysis

Data gathering and analysis were organized according to the HRIA tool. All selected papers were abstracted and entered into a matrix describing study design and findings (see Table 2). Findings were then categorized into a framework of themes reflecting the areas covered (health and its determinants). This provide the structure of the review. The assessment was not quantitative; rather, it was simply heuristic to illustrate how right to health is affected by economic sanctions (according to HRIA framework). Based on the findings, related obligations of Iran and other states about effects of sanctions on Iranians’ right to health were extracted. In the case of incompatibility of a certain policy with human rights obligations, HRIA suggests several options: termination or amendment of the policy, insertion of safeguards, and adoption of compensation measures or other modification measures.^37^

**Table 2:** Main Findings of the Review of Literature

	**Authors**	**Method**	**Relevant Findings/Message**	**Humanitarian Effects**		
				**Healthcare**	**Medicine**	**Determinants of Health**
1	Baradaran-Seyed and Majdzadeh^39^	Review of reports of Iranian Academy of Medical Sciences, Iranian Medical Council and UN Secretary General	Sanctions against Iran’s financial system made medicine inaccessible.		*	
2	Butler^40^	Interview with health professionals	- Economic sanctions against Iran caused acute shortages of medicines, vaccines and key medical supplies. - Humanitarian exemptions on food and medicine did not work.	*	*	*
3	Cheraghali^41^	Observations from Iran’s pharmaceutical market	Sanctions in foreign trade, financial and banking services against Iran resulted in inaccessibility of life saving medicines and weakened national health sector and affected ordinary people.	*	*	
4	Ghiasi et al^42^	Analysis of the collected data from a group of pharmacies	Imported and locally produced asthma medicines were not accessible in community pharmacies of Tehran during 2012-2013 in which sanctions against Iran were intensified.		*	
5	Golzari et al^43^	Observations of pharmaceutical market	Economic sanctions against Iran had led to shortages of patented and generic cancer drugs while this country has the highest incidence of cancer in the Middle East.		*	
6	Gorji^44^	Observations of pharmaceutical market	- Economic sanctions against Iran influenced all branches of Iranian economy and affected vulnerable patient. Unavailability of medicine and raw materials for domestic pharmaceutical companies and medical equipment for hospitals and unaffordability of medicines were big challenges of Iran during sanction’s period. - The weakened medical infrastructure decreased the ability of the health system to provide services even in medical emergencies. - Establishing uniform operational criteria and definitions for exemptions of medicine and medical products from sanctions in the future sanction regimes is necessary.	*	*	
7	Hajizadeh and Nghiem^45^	Investigating health system of Iran from financial, utilization, and quality perspectives	Quality of hospital care is different among different provinces of Iran. Economic sanctions seem to have negative impact on Iran's healthcare delivery system.	*		
8	Hosseini^46^	Observation of pharmaceutical market of Iran	- Because of sanctions on foreign trade, financial and banking services, Iran faced major difficulties for importing medicines and medical instruments. - Quality, accessibility and affordability of medicines decreased during sanction’s period. - In a public health prospective, ordinary people and patients paid the cost of economic sanctions. Appropriate measures should be taken by international community to facilitate access to medicine. To facilitate the process of medicine’s importation, defining a white list of Iranian pharmaceuticals companies and their foreign counterparts is necessary.		*	
9	International Campaign for human rights in Iran^31^	Review of scholarly material and journalistic accounts and interviews with a cross-section of Iranians	Economic sanctions against Iran and the country’s policies resulted in a significant economic decline and deterioration of living standards of Iranians. Sanctions decreased affordability and accessibility of healthcare and medicine.	*	*	*
10	International Institute for Peace, Justice and Human Rights^47^	Interviews with health professionals, patients, health services managers and pharmacists	Sanctions on Iran have had destructive effects on healthcare system, Iranian’s lives and quality of life. Because of sanctions on banking system, import of medicines has become difficult. These are violations of the basic human rights.	*	*	*
11	Karimi and Haghpanah^48^	Examining the effects of sanctions on access to healthcare from patients’ point of view and related physicians	By adversely influencing accessibility of medicines, sanctions had considerable effects on public health and health of patients with thalassemia and hemophilia.		*	
12	Kheirandish et al^49^	Media analysis	Negative effects of the sanctions on access to medicines in Iran after the sanctions of 2012 is proved.		*	
13	Mohammadi^50^	Interview with clinicians	Economic sanctions against Iran affected the availability of essential and life-saving medicines and forced patients to seek their medicines from an unregulated black market.		*	
14	Moret^51^	Analyses of semi-structured interviews, official discourse and case studies	Economic sanctions negatively impacted the health of ordinary citizens in Iran and Syria through factors such as difficulty in access to medicine and food.		*	*
15	Namazi^32^	In-depth interviews with Iranian importers, manufacturers, and distributors of pharmaceuticals and medical equipment as well as their Western counterparts	Economic sanctions against Iran have had severely affected the availability, accessibility and quality of medicines in Iran.		*	
16	Roshan et al^52^	Review of literature on the situation of some health indicators before and during sanctions’ period	Economic sanctions have had negative effects on people's health particularly in the field of medicine and treatment, air pollution and the environmental health.	*	*	
17	Setayesh and Mackey^53^	Review of key characteristics of the Iranian drug shortage	73 scare medicine were closely tracked with the disease burden of the country. 44% of these medicines were classified as essential medicines by WHO. A vast majority of these medicine were exempted theoretically from the sanctions.		*	
18	Shahabi et al^54^	Literature review	Iran's NCCP has substantial deficits, including a serious shortage of medicines for cancer care. Sanctions exempted medicines and food, but lead to disruption of health services through complications in shipment or lack of foreign currencies.		*	*
19	Iranian Society of Atherosclerosis^55^	Observation of pharmaceutical market	There was an acute shortage of essential medicines for patients with cardiovascular disease in Iran in 2012.		*	
20	Takian and Kazempour-Ardebili^56^	Review of Iranian National Action Plan for the Prevention and Control of NCDs and WHO framework	There are many obstacles hindering the achievement of the targets of the Iranian National Action Plan for the Prevention and Control of NCDs including lack of financial resources. Sanctions exacerbated the situation. Safeguarding peoples’ health and well-being in the post-sanctions era is necessary.	*	*	
21	UN Economic and Social Council^57^	Review of reports and observations	The number of street and working children has increased from 2009-2013. They have limited access to healthcare and education.	*		*
22	UN General Assembly^58^	Review of reports obtained from local newspapers and institutions such as UNICEF and observations	Sanctions adversely affected standard of life of Iranian’s particularly of children and elderly people. Medicine for the treatment of diseases such as cancer, heart disease, thalassemia, HIV/AIDS, hemophilia and multiple sclerosis were not accessible from 2012-2013. Also, humanitarian exemptions for food, medicines and medical supplies could not meet their intended purpose.		*	*
23	UN Refugee Agency^59^	Review of reports and observations	The sanctions affected adversely the life of refugees in Iran, and operational and humanitarian assistance costs of UN.			*
24	UN Secretary-General^60^	Review of reports and observations	Unemployed women and female-headed families have been vulnerable to country’s economic decline of recent years in Iran. Since they are economically dependent to and more probable to face poverty.	*		*
25	UNICEF^61^	Review of reports and observations	Tightened unilateral sanctions against Iran have adversely affected the environment, public health and socio-economic determinants of health of ordinary people, especially children. In recent years, the number of working and street children has increased. These children have limited access to health services and education.	*		*
26	Zare et al^62^	Spline and quantile regression techniques	Income elasticity is lowest for the poorest Iranians in urban and rural areas Economic sanctions on Iran have the potential to disrupt government-subsidized healthcare services.	*		
27	Kebriaeezadeh et al^63^	Systematic literature review	Iranian pharmaceutical market has undergone great growth till 2011. Before sanctions, Iran’s national pharmaceutical industry could provide essential medicines to patients.		*	
28	Hashemi-Meshkini^64^	Literature review	Financial and trade sanction revealed the weaknesses of Iran’s domestic pharmaceutical industry in proving medicines and medical devices in political and international crisis.		*	
29	Kheirandish et al^65^	Review of published reports and articles	Iran faced major challenges in the provision of adequate access to medicines during sanctions of 2010–2014. Economic crisis might lead to changes of national priorities for investment and expenditure and reduce government’s available resources, and thus may affect the health system and access to medicines.	*	*	
30	Asadi-Pooya et al^66^	Retrospective chart review study of drug adherence of patients with epilepsy	Unavailability and unaffordability of medicines resulted in poor drug adherence. Shortage of medicines and increase of the price were directly associated with intensifying of economic sanctions against Iran. These sanctions brought a bout considerable socio-economic hardship for Iranians.	*	*	
31	Duttagupta et al^67^	Examining healthcare financing and market access implications of pharmaceuticals in light of the introduction or removal of sanctions.	Lifting of economic sanctions on studied countries including Iran will have a positive impact on innovative pharmaceuticals, from perspectives of market access and technology transfer.	*	*	
32	Deilamizade and Esmizade^68^	Interviews with drug users, questionnaires, participants’ observations, and statistical analysis of the existing data were used.	Because of economic sanctions, the price of goods and services including drugs has increased in Iran. Major changes in the drug use patterns and an increase in use-related harms of drug are expected in near future.			*
33	Massoumi and Koduri^69^	Interviews with physicians and review of literature	Sanctions caused limitations in the import of medicines (pharmaceutical ingredients and finished products) and access to patented ones. The quality of substituted medicines was not satisfactory while these scare medicines were not affordable for some groups of patients.		*	
34	Ahmadi and Meskarpour Amiri^70^	Review of literature	Study of targeted countries by sanctions including Iran showed that through limiting access to minimum basic needs, economic sanctions can threaten public health especially health of the mothers and children of poor families. Reduction of target countries’ revenues can lead to reduction of government capacity to finance healthcare system and increase of the share of households in healthcare costs and adversely affect access to healthcare services.	*		
35	Gordon^71^	Review of literature	Food security and access to healthcare and education were not supposed to be undermined by economic sanctions against Iran. The sanctions influenced every sector of Iran’s economy and public services which were necessary for well-being of the whole population specifically women who are most economically vulnerable. Female heads of households faced much stress trying to feed their families, obtain medicines, and buy necessary goods. In the sanctions period, unemployment and bankruptcies increased substantially.	*	*	*
36	Dizaji et al^72^	Comprehensive set of VAR models	Social impact of economic sanctions against Iran may extend beyond the sanction period because “the costs of imposing sanctions exceed the benefits of lifting sanctions.”			*
37	Farzanegan et al^73^	Examining the macroeconomic and household welfare consequences of oil sanctions in Iran by using social accounting matrix and developing a computable general equilibrium model	Iranian economy and households are affected enormously by economic sanctions. The welfare of all income groups of households in urban and rural areas has declined.			*
38	Kermani^74^	Analyzing the conceptual framework of political economy	Sanctions adversely affected people’s livelihood. It is ignoring basic human rights of Iranian’s particularly the rights to life, health, access to medicine and development. Promoting justice by discriminatory punishment of innocent people is impossible.	*	*	*
39	Chenoy^75^	Review of reports and papers	Smart sanctions against Iran has severely impacted on socio-economic pattern and the lives of ordinary people. Inflation and shortages have led to high costs of food prices. The decline in women's status, job security and opportunities coincide with sanction regime.			*
40	Rezapour et al^76^	Concentration Index on inequality	In recent years, out-of-pocket payments for health increased while the capacity of households to pay decreased. The poor spend a greater portion of their capacity-to-pay for healthcare, in comparison to the rich. Sanction-borne inflation in economic and health sectors has caused financial crisis. Supporting the poor and decrease of out-of-pocket must be considered.	*		
41	Ebrahimi et al^77^	Literature review	Sanctions resulted in deprivation and restriction of Iranians in enjoyment of their rights to a standard of life, health, education and development.	*		*
42	Menezes^78^	Theoretical conceptual approach	Because of economic sanctions, health system of Iran faced difficulties in the import of medicines and medical equipment.		*	
43	Neuenkirch and Neumeier^79^	Nearest neighbor matching approach	US sanctions have led to larger poverty gap in sanctioned countries including Iran compared to their nearest neighbors.			*
44	Palaniappa^80^	Comparative study using review literature	Iran’s health indicators used to be one of the best in the Middle East. The sanctions against Iran have had notable humanitarian implications, specifically on economic growth and health sector. The sanctions have decreased oil revenue and immensely destroyed economy of Iran. Unemployment rate, inflation, and commodity prices increased. It caused major shortages of medicine since the organizations with proper licenses were unable to find third-country banks for importing medicine and food. Due to the rise in food costs and general inflation, many lower and middle class families could not afford food.	*	*	*
45	Sha’bani et al^81^	Content analysis of library resources and Internet data	Socio-economic status of Iranians is influenced adversely by the sanctions in recent years. Iran is facing a great deal of different challenges such as unemployment, inflation and depression, immigration, and marriage problem, brain drain and economic downturn.			*
46	Asadi et al^82^	Content analysis and comparative study	Studying Iran showed that sanctions with a large economic effect on the target country can have severe public health consequences which are very much similar to the effects of major military conflict.‏ Using resistive economy might be helpful in this situation.	*		
47	Bastani et al^83^	Content analysis with an inductive approach applying a 5-stage framework analysis (familiarization, identifying a thematic framework, indexing, mapping, and interpretation)	Sanctions can influence the final price of domestic medicines, production quality and the hidden prices of imported medicines. In order to improve access to medicine in Iran, affordability of medicines, effects of exchange rate fluctuations on the cost of pharmaceuticals, influence of sanction on the final prices of pharmaceuticals, efficiency and patient's ability to cooperate in payment should be taken into account.		*	
48	Portela^84^	Analysis of the design of different categories of sanctions instruments	In Iran, sanctions affected the economy, healthcare and environment and caused a decline in the living standards of the population. It made acquisition and distribution of medical and pharmaceutical supplies difficult while legislation exempted the importation of humanitarian items from sanctions. No plan for monitoring of impacts of sanctions against this country was devised.	*	*	*
49	Dizaji^85^	Impulse response functions and variance decomposition analysis	The decreases in Iran’s revenues limited the government financial capability for financing health, education and social security and payment to its employees which damaged the Iranians’ standard of life.	*		*
50	Taghdisinejad and Allahmorad^86^	Interviews with the elite using Delphi	Sanctions have led to increase of inflation, decrease of government’s revenues, public investment, employment, Job Security and stability as well as households’ income and purchasing power and government’s abilities to support vulnerable groups.			*
51	Nematolahi et al^87^	General equilibrium pattern	Economic sanctions against Iran and change of Iran’s policy about subsidized food have increased food price and decreased purchasing power of households and food security.			*
52	Mostafavi et al^88^	Hsiao causality procedure	Economic sanctions influenced production models from clean production techniques to pollutant ones and led to more use of old technologies and air pollution.			*
53	Mousavi et al^89^	Content analysis of international human rights laws and review of reports and literature	- Sanctions against Iran influenced the livelihood of Iranians and resulted in more poverty and less welfare. - Because of economic downturn, inflation, decrease of households’ purchasing power, some groups of the population cannot access necessities of life such as food and shelter. - Particularly the sanctions resulted in violation of Iranian’s right to health by adversely effecting accessibility of medicine and medical devices, increasing healthcare costs, decreasing government’s financial ability for support of the poor and limiting import of quality gasoline.	*	*	*
54	Mashhadi and Rashdi^90^	Literature review	Iranians’ right to a healthy environment is violated by recent sanctions on import of gasoline and energy sector and prohibition of related knowledge and technology.			*
55	Marzban and Ostadzade^91^	Extension of a generalized growth pattern despite a random exchange rate boycott	Since the economy of Iran is dependent to oil revenues, the sanctions resulted in decrease of Iranians’ welfare.			*

Abbreviations: UN, United Nations; NCCP‏, National Cancer Control Program; WHO, World Health Organization; UNICEF, UN Children Found; VAR, vector autoregressive; NCD, non-communicable disease.

## Results

Economic sanctions have the potential to adversely affect the welfare and health of targeted populations. In the case of Iran, the results of the literature review indicated that the sanctions adversely affected affordability, accessibility and quality of health services and medicine and exacerbated the living standards of Iranians. In the next part, the effects of sanctions on Iranians’ health and its determinants are explained. This is followed by a discussion on the obligations of Iran and other countries about the humanitarian consequences of the sanctions.

## Part 1) Iranian’s Enjoyment of Their Rights in Sanctions’ Period

### Effects of Economic Sanctions on Iranians’ Standard of Life

After gauging the humanitarian impacts of sanctions on Iranians’ lives, particularly their access to food and medicine through extensive news reports and reports of UN General Secretory, the United States permitted its companies to sell selected medicines and medical supplies to Iran without requesting a license from the Treasury’s Office of Foreign Assets Control at the end of 2012.^92^ Also, through the Joint Plan of Action, a channel for humanitarian support was established between Iran and six other countries in November 2013.^93^ However, these exemptions of humanitarian trade, did not guarantee Iranians’ access to food, medicine and medical equipment. Since, limitations on trade, banking and financial system and shipment (mentioned before), made the transferring of any goods, including those exempted ones to Iran, extremely difficult and expensive. UNICEF describes Iran of 2012 as a country under tight unilateral economic sanctions which are adversely affecting the environment, public health and the socio-economic determinants of health of ordinary people, especially children.^61^ In the UN report on October 5, 2012, General Secretary, Ban Ki Moon stated that “The sanctions imposed on Iran have had significant effects on the general population, including an escalation in inflation, a rise in commodities and energy costs, an increase in the rate of unemployment and a shortage of necessary items, including medicine[…] The sanctions also appear to be affecting humanitarian operations in the country[…] Even companies that have obtained the requisite license to import food and medicine are facing difficulties in finding third-country banks to process transactions.”^94^

Economic sanctions diminished Iran’s economy considerably; from 2012 to 2014, GDP per capita fell dramatically by 35% (Figure 1).^33^ While the value of national currency declined by 80% during 2011-2013. In 2012, the overall inflation rate of consumer price index was 36% and 41.4% respectively in cities and rural areas.^95,96^ Sanctions influenced all aspects of Iran’s economy including public services that are necessary for welfare of the population.^71^ They also contributed to increase of the rates of inflation and unemployment (Figure 3).^71,80^ Fall of Iran’s revenues led to decrease of government’s resources to pay its employees’ salaries.^85^ Almost all Iranian manufactures were hit by economic sanctions. Operating with partial capacity, they could not pay timely wages (which were below poverty line) and had to fire many workers that deteriorated the living conditions of workers and their families considerably.^31^ As a result, purchasing power of population decreased (Figure 3).^34,86^ Minimum wage decreased by more than 50% during 2010 to 2012 (Table 1).^35^ Sanctions influenced the socio-economic status of the population, increased poverty and widened the income gap between different groups of Iranian society and decreased welfare of the most vulnerable to a great extent.^66,73,91,97^ It was estimated that about 11% of Iranians were living below absolute poverty line and 30% under relative poverty line in 2016.^98^ The population of the poor in rural and urban areas have been respectively 15% and 13% in 2012.^99^ By limiting revenues of the government, sanctions decreased the capabilities of the state to support the poor.^89^ In 2013, the UN Special Rapporteur on the situation of human rights in Iran highlighted the dramatic effects of sanctions on Iranians’ standard of living.^58^ Sanctions affected people’s standard of living and violated their rights to education, health and development.^77,84,85^

Sharp declines in the value of Iran’s currency and the country’s dependency on the import of food and its related industries and the subsequent change of Iran’s policy on subsidized food caused a sharp rise in the price of food. As a result of the significant increase in unemployment, many Iranians reduced household expenditures by consuming less quality and quantity of food.^54,80,87,89^ Moreover, along with the deterioration of the economic situation of families, more children left school to work, and got married to lower the financial burden on their parents. In recent years, the number of working and street children has also increased dramatically. These children have limited access to health services and education.^57,58,61^ According to Statistics Center of Iran, formal child marriage increased more than 20% during 2012-2014.^100^ In the province of Isfahan, the number of street children has increased by 120% during 2015-2016.^101^ There is no official data about the number of street children in Iran. Statistics provided by different institutions range from two to seven million street children. Based on the reports of National Statistics Center of Iran, 1.7 million children work in Iran.^102^ It was estimated that this number has been about 700 thousands in 2009.^103^ This situation exposed children to violence, drug addiction, HIV infection and harmful work such as selling of drugs. There are cases of child trafficking; even though it is considered a crime by the laws of Iran.^104^

Furthermore, in the sanctions period, women faced more socio-economic hardships; job security and opportunities for women decreased.^75^ The effects have been more immense on women who were economically dependent to the family or were heads of their family.^71^ A considerable number of Iranian women are unemployed and economically dependent to their spouses and children, and vulnerable to country’s economic decline. Particularly female-headed households faced more poverty and often could not afford nutritious food and healthcare in 2013 and 2014.^45,60,105^ In addition, Iran is facing a new phenomenon of street women and significant increase of addicted women and sex workers. Chronic poverty is one of the main reasons of entering the illegal market of sex work in Iran.^106^ Furthermore, decline of financial ability of working age people exacerbated the living situation of the elderly too. Old Iranians usually are financially dependent to their children; in recent years, the number of homeless elderly people has increased too.^58^ A high percentage of young Iranians, including educated ones, are unemployed and living in poverty. In 2015, 57% of unemployed people were at the age group 15-29. The unemployment ratio of this age group has increased by 2.6% comparing to 2014.^107^ The rate of mental conditions such as depression and stress is high among unemployed young people.^108^ Depression specifically increased after intensifying sanctions against Iran due to deterioration of people’s economic situation.^81^ Furthermore, Iran is one of the biggest hosts of refugees and asylum seekers in the world. Most of the refugees are from Afghanistan and Iraq. The sanctions affected adversely the life of refugees in Iran, and operational and humanitarian assistance costs of UN.^59^

### The Impacts of Sanctions on Iranians’ Right to Health

Rights to healthcare and social security are guaranteed by the Article 29 of Iran’s Constitution. In recent years, Iran has provided free primary healthcare throughout the country and improved the quality and quantity of health services to a great extent. However, over the years, financial accessibility of health services has continuously declined; while health insurance system of Iran has not provided a universal coverage yet. Most of uninsured people are from the lowest income groups. Patients’ share of healthcare expenditure was 52% of total health expenditures and 88% of private health expenditures in 2012. The government and insurance companies paid the rest.^109^

In 2012, with intensifying sanctions on Iran, inflation rate in health sector was 44.3% and 45.6% respectively in cities and rural areas.^110^ Insurance companies reacted to the inflation by decreasing their list of services. It resulted in the increase of patients’ share of health services and ended in withdrawal of healthcare and more reliance on self-treatment. Several studies showed increasing tendency of Iranians to self-medication; they have warned about adverse effects of reliance on self-treatment in Iran.^111-113^ Still services of public health facilities were cheaper than private ones; but they were overcrowded and came with shortages and long waiting lists. In response, a few well-off patients traveled to other countries to get healthcare. Some other patients, especially with terminal and incurable illnesses, withdrew from health services due to inability to pay.^47,114^ Sanctions with a large economic effect on a target country (similar to Iran’s case) can have severe public health consequences which are very much similar to the effects of major military conflict.^83^ Sanctions on Iran had the potential to disrupt government’s subsidized healthcare.^62^ Economic sanctions decreased the revenues of Iran’s government and its ability to invest on health, education and social security of Iranians.^84,85^ Also, they forced the government to change the priorities of national investment from certain public responsibilities, such as supporting the poor.^65^ Therefore, the share of people of health services costs increased that adversely affected access to healthcare.^70^ Low income groups were more vulnerable to the effects of the sanctions. The poor paid bigger proportion of their income on healthcare.^76,84,85^ In 2013, Iran enacted a law to reform heath system; one part of this law requires the government to cover at least 90% of hospital services’ costs by public funds.^84,115^ But still every year 1% of the population fall below poverty line due to catastrophic health expenditures.^116^

By limiting access to necessities of life, sanctions against Iran endangered public health particularly health of mothers, children and the poor.^70^ Economic sanctions have had other impacts on health of people in Iran. For example, due to ban on fuel trade and related production knowledge and technology, locally produced poorly refined fuel was substituted that had the main role in polluting the air all over the country.^52,89,90^ Economic sanctions influenced production models from clean production techniques to pollutant ones and led to more use of old technologies and air pollution.^89^ About 45 000 deaths in one year and increase of lung cancer cases among children were reported to be linked to air pollution in Iran.^61^ Another example is ban of selling air craft parts to Iran that resulted in unsafe flights and endangered lives of people. The same happened to automobile industry equipment. The sanctions also might endanger mental health of people because of continuous signals of threats and deteriorated living situation. Recently, the rates of mental diseases and drug addiction and cases of suicide among Iranians have increased considerably. According to the report of Iran’s Ministry of Health and Medical Education, the rate of mental diseases has increased 4% in recent four years. The suicide rate increased 7.6% during 2012-2013.^117,118^ Unemployment and financial distress are two main causes of mental conditions in Iran.^119^ A study in the province of Ghazvin showed that unemployment, inflation and welfare inequality had a meaningful relation with suicide rates.^120^ Another example is the impacts of economic decline on drug addicts and their families. While the price of medicines for treatment of drug addiction and drugs itself increased, having less financial means, drug users tend to decrease spending on their family’s life and use cheaper substances.^68,121^ New substances are associated with high risk methods of use such as injection and high risk sexual practices and more acts of violence and can have long term effects on body organs.^122^

The harms of economic sanctions to health indicators can remain hidden for several years and become evident in a longer period. As an example, unavailability of financial resources caused by sanctions is known as an obstacle to meet the goals of Prevention and Control of Non-communicable Diseases Program in Iran.^56^ Sanctions can have more direct and immediate adverse effects on health by limiting the availability and accessibility of medicine. In theory, medicine exempted from sanctions against Iran, but in practice, access of patients to quality medicine became limited due to the sanctions of 2012. The report of UN Special Rapporteur on the situation of human rights in Iran 2013 indicated that “humanitarian safeguards in the form of exemptions for foodstuffs, medicines, chemicals for the production of medications and medical supplies are failing to meet their intended purpose. Reports indicate that financial sector sanctions effectively frustrate the purpose behind humanitarian exceptions. They also stress that the supply of advanced medicines, which treat the most serious illnesses, are particularly affected. Advanced medicines are produced primarily by firms based in Western countries and are subject to 20-year patents, rendering it impossible to substitute products from an alternative source.” The ineffectiveness of humanitarian safeguards is apparent in Iran.^58^

### Effects of Sanctions on Right to Medicine

Universal right to safe, effective and affordable medicine is a fundamental element of right to health; states should consider this right in their international agreements.^123^ By having a national generic policy, a network for provision and distribution of medicine and an overarching pricing system for medicine around the country, Iran had a satisfactory level of access to medicine and medical instruments before comprehensive sanctions.^49,63^ Depended to the type of services, insurance companies cover 70%-90% of retail price of medicines. Iran produces about 96% of all the medications of its pharmaceutical market in the terms of number and volume. But, the value of imported medicine is about 40% of whole market’s value.^46^

With the tightening up of sanctions in 2012, the situation changed; government faced difficulties in provision of medicine; locally produced and imported medical equipment and medicines started to be scarce.^66,67,78,80^ Medicines were not subject to sanctions, but limitations on licensing, purchase and shipment of goods to Iran made the import of medicines difficult. Also, Iran is dependent to import of pharmaceutical’s raw materials, and production and quality control technologies which were not exempted from sanctions. Therefore, the sanctions crippled domestic pharmaceutical industry and disrupted the production and quality of generic medicine too.^51,55,69^ International pharmaceutical companies (and banks) were reluctant to deal with Iran because of the potential threat of secondary sanctions and difficulties of receiving payment.^50^ Being cut off from international banking network, Iranian government had to pay cash in advance that was very difficult, if not impossible, for mass imports of medicines. Moreover, the shortage of foreign currency and decline of country’s currency value made medicines expensive.^69^ In addition, a complex process of providing banking facilities to importers and extremely lengthy process of importation caused further shortages. Therefore, the cheapest medicines such as contraception pills and simple medical instruments such as vaccines, sutures and endoscopy instruments were not available by 2012.^39^ These all indicate that pharmaceutical system of Iran has been weak in provision of medicine in emergency and unusual situations such as economic sanctions.^64^

Following multilateral sanction as of 2012, the import of medicines and raw medical materials fell by 30%-55%^58^ and the average shortage of medicine from less than 30 in type reached to 144.^52^ Forty-four percent of scarce medicines were classified as essential medicines (minimum requirement for a functioning health system) by WHO. A vast majority of these medicines were supposed to be exempted from the sanctions.^53^ Access to nuclear medicines and radiotherapy pieces for diagnoses and treatment of cancer were completely cut off, since they were in the list of sanctions due to the possibility of military usage.^43,47^ Several studies showed that access of about 6 million patients with life-threatening diseases such as asthma, thalassemia, hemophilia, chronic diseases, blood disorders, multiple sclerosis and HIV/AIDS to their medicines was limited.^44^ In addition, anti-rejection transplant medicines, and kidney dialysis instruments were scarce by 2013.^42,48^ Domestically produced replacements were scarce and not effective enough. Unavailability and unaffordability of medicines resulted in poor drug adherence.^66^ A number of deaths due to a lack of access to medicines were reported in 2012.^55,124^ Furthermore, the shortage of medicines reduced the ability of health system to provide services even in emergencies; suspended operations were serious problems following sanctions 2012.^48^ Moreover, Iran does not produce drugs for eradicated diseases and raw material for antibiotics that can threaten universal public health. For example, Iran could not produce BCG vaccine till 2015. Moreover, shortages of medicines and medical equipment needed for diagnosis and treatment of some diseases might change the county’s overall disease burden; the number of deaths by non-communicable diseases (including cancer, diabetes mellitus, cardiovascular diseases, digestive diseases, skin diseases, musculoskeletal diseases, and congenital anomalies) has increased in recent years in Iran.^52^

To compensate 30%-46% fall of medicines’ import from the United States and EU during 2011-2012, Iran increased its purchase of medicine and medical equipment from countries that did not ban oil trade with Iran. From 2012, the purchase of medicine from China and India increased respectively 2 and 5 times.^110^ However, the alternative medicines usually had lower quality and limited effectiveness than equivalents from Europe or United States.^32,69^ Moreover, medicines have to be registered and their safety and effectiveness must be approved by Iran’s Medicine and Food Organization to be allowed to be produced, imported and distributed in Iran.^125^ This process takes several months. In response to the shortages, Iran allowed medicines which were approved by US Food and Drug Administration (FDA) or European countries to be imported without assessment and national approval. But this resulted in major side-effects including intolerance by the patients to a change in a long term treatment.^32^ On the other hand, due to the absence of official supplies in health facilities and pharmacies, smuggled medicines increased in the local market of Iran. They were often out of date, of poor quality, contaminated, or spoiled by climate extremes. They were also sold at a price several times higher than the official price. It was also difficult to know whether they were counterfeit or real.^41^ In 2013, following eye surgery, 22 patients got serious infection in their eyes and were in danger of losing their visions because of using a non-standard ampoule.^126^

Economic sanctions also made medicines financially inaccessible from 2012 to 2013. The increase in medicines’ price was 50%-75% during this period.^44^ The price of most medications has not been a problem until recent years in Iran. To guarantee access of people, Iran provides a subsidy for selected medicines. Usually, the amount of subsidy is determined at the end of each year based on the estimation of medicines’ price and country’s revenues in the coming year. The sharp fall of revenues and in the value of country’s currency was not predicted in 2012. After facing shortages, Iran allocated more financial resources and foreign currencies for the import of medicines and could establish companies in neighboring countries to use their banking system for purchasing medicine. As a result, many key medicines are now available; but still it is expensive for some diseases. The situation is more difficult for the poorest people who are not insured. In this condition, some health services such as dental care has become a privilege which is inaccessible to the working and middle classes of population.^40^

### Human Rights Impact Assessment of Economic Sanctions Against Iran

The results of the literature review concur that economic sanctions against Iran have resulted in depriving Iran’s population of their economic and social rights, specifically the right to health and its determinants. About 80% of the studied papers indicated that sanctions adversely affected access to health services and related products. Sixty-three percent of papers confirmed the inaccessibility and affordability of medicine for the population. Moreover, 56% of the papers showed that sanctions affected the determinants of health such as environment health, employment and access to food. The sanctions on Iran caused a fall in the country’s revenues, devaluation of the national currency, and increased inflation and unemployment. These all resulted in a deterioration of people’s overall welfare and lowering their ability to access the necessities of a standard life such as nutritious food, healthcare and medicine. Also, sanctions on banking and financial system and shipment led to scarcity of quality lifesaving medicines. The impacts of sanctions were more immense on lives of the poor, patients, women and children. According to HRIA tool, the economic sanctions against Iran have had a significant humanitarian effect; therefore they should be reconsidered. To place pressure on Iran to limit its nuclear activities, other measures should be used instead that do not have such effects. While international peace should be respected, human rights obligations cannot be forgotten. Review of the obligations of Iran and international community about Iranians’ right to health is essential when considering compensation measures.

### Part 2) States’ Obligations in Sanctions Period

To protect people in the sanctions period, imposing countries, international community and target country have human rights obligations. The fundamental part of these obligations is that everybody should enjoy his/her rights without discrimination of any kind.

#### Targeted Country’s Obligations in the Sanctions Period

Review of Iran’s response to the sanctions indicates that this country has not been prepared for the sanctions, delayed in responding to shortages and could not manage the situation appropriately. Also, bureaucratic constraints, corruption and inefficient resources management contributed to the deterioration of people’s enjoyment of their basic rights.^51,71^ In the period between announcement and implementation of the sanctions, Iran should have provided a national policy with suitable measures to prevent suffering of people from the adverse effects of sanctions and to ensure everyone enjoys his/her basic rights including right to health. The failure to take appropriate steps towards progressively realization of right to health and to enforce related laws is in contrast with international human rights obligations. Moreover, Iran should have prevented third parties; black market dealers, pharmacies and health facilities that provided unsafe medicines, as well as smugglers who sent scarce medicine to neighboring countries from violation of people’s rights. Failure to regulate third parties’ activities is against the right to health.^20^

Countries targeted by sanctions should respect international peace, security and human rights obligations to alleviate a humanitarian crisis.^127^ In the sanctions period, the government of the target country has human rights obligations. People’s lack of access to nutritious food, primary healthcare, basic shelter and education indicates that the country has failed to discharge its obligations under ICESCR. The State is required to monitor human rights situation and utilize the maximum resources available to eliminate the suffering with low cost programs, international assistance and cooperation. Moreover, even in severe resource limitations, vulnerable groups of the population such as children and the poor must be protected. Non-compliance with the core obligations of right to health (access to health facilities, products and services and minimum essential food, basic shelter, sanitation and safe water) cannot be justified under any circumstances.^20^ The CRC also states that children are entitled to human rights; parents without enough means should be supported to provide a standard of life for their children. Governments should prohibit child marriage, child trafficking, child work, violence against children and engagement of children in drug selling. The needs of immigrants’ and asylum seekers’ children should be taken into account too.^128^ Generally, states have the obligations to respect, protect and fulfill right to health. They must ensure that everyone, without any kinds of discrimination, enjoys this right. In all times, including the period of sanctions, states are required to provide available, accessible, acceptable and good quality facilities, goods and services related to health and its determinants to everyone. In addition, they are required to use all necessary means, the maximum of their available resources and to adopt appropriate legislative, administrative, budgetary, judicial, promotional and other measures towards the full realization of right to health.^20^ There is the possibility to seek international assistance for realization of the right to health too.

#### International Community’s Obligations

The international community has two kinds of obligations in relation to the right to health. First, in cooperation with international organizations, all countries are required to provide conditions at international and regional levels to ensure everyone enjoys the right to the highest attainable standard of physical and mental health. They should help developing countries to progressively realize this right through the establishment of effective and integrated health systems with adequate, affordable and reliable and good quality supply of medicines.^129^ Second, they should avoid violation of this right and prevent third parties such as international organizations and groups of countries also violating this right.^20^ According to the UN Charter 1945, Security Council may decide what measures including sanctions should be employed to maintain or restore international peace and security.^16^ Generally, economic sanctions which are imposed by some countries against the others are inconsistent with the Charter’s basic principles of equality and dignity of every human being. Resolution 39/210 of UN General Assembly 1984 states:

Developed countries should refrain from threatening or applying trade and financial restrictions, blockades, embargoes, and other economic sanctions, incompatible with the provisions of the Charter of the UN and in violation of undertakings contracted, multilaterally and bilaterally, against developing countries as a form of political and economic coercion that affects their political, economic, and social development.^130^

The Vienna Declaration and Program of Action 1993 requires the states “to refrain from any unilateral measure not in accordance with international law and the Charter of the UN that creates obstacles to trade relations among States and impedes the full realization of the human rights set forth in the Universal Declaration of Human Rights and international human rights instruments, in particular the rights of everyone to a standard of living adequate for their health and well-being, including food and medical care, housing and the necessary social services.”^131^ Adoption of laws interfering with the enjoyment of right to health, failure to take into account the legal obligations related to this right in bilateral and multilateral agreements and also failure to regulate activities of third parties in order to prevent them from violating right to health are violations of the ICESCR obligations. Imposing embargos and other measures that restrict the supply of another state with adequate medicine and medical equipment should be refrained. The Committee on ESCR prohibits restriction on these goods as a tool for political and economic pressure. States must respect the enjoyment of right to health in other countries by refraining from denying or limiting the access of people to healthcare and medicine. They must ensure that their international agreements do not adversely impact upon this right. The states who are members of international and regional financial institutions should consider protection of the right to health in their credit agreements and lending policies.^20^

States are responsible legally for their policies that violate human rights of people beyond their borders and for the policies that support this action by third parties. According to paragraph 39 of General Comment no 14 ICESCR, “to comply with their international obligations in relation to article 12, States parties have to respect the enjoyment of the right to health in other countries, and to prevent third parties from violating the right in other countries, if they are able to influence these third parties by way of legal or political means, in accordance with the Charter of the UN and applicable international law.”^11^ Countries that impose, maintain or implement sanctions should take immediate steps to respond to the suffering experienced by people in targeted countries.^20^ This can be done by facilitating the delivery of necessary items for life and health such as medicine, food and medical equipment. The states imposing sanctions should carefully assess the effects of their policies and international agreements on the health of people in the target country. Subsequently, they must develop policies and laws to alleviate the negative impacts of their agreements on the human rights of population in the target country.^132^ A country’s population should not be deprived of their basic ESCRs due to any determination that their leaders have violated international peace and security norms.^20^ The international community must protect the core content of the ESCRs of targeted population. UN bodies should observe the situation of human rights and implement humanitarian and human rights laws. Otherwise, basic principles underpinning international law such as equality of all human beings will lose credibility. In addition, the Security Council should alleviate sanctions regimes in order to eliminate the humanitarian effects of sanctions on target population and ensure that they have access to food, medicines and other necessities of preserving health.^127^ It seems that regulations on humanitarian exemptions are formulated imprecisely and confusing; they lack standards and an impact monitoring system. Also, exempted goods and their distribution are poorly understood and interpreted, and often the urgent delivery of humanitarian supplies is blocked.^7,133^ These all have major implications on the basic rights of the population of target country.

## Discussion and Conclusion

According to the results of HRIA, economic sanctions against Iran have resulted in violation of Iranians’ right to health. There is incompatibility between obligations derived from economic sanction agreements and human rights. In this case, HRIA suggests several options: termination or amendment of the policy, insertion of safeguards, and adoption of compensation measures. The main principle of this assessment is that no policy at national and international level should breach international human rights laws. This tool, at first step, suggests that such policies should be stopped. However, it does not mean that countries should not face any limitation if they threaten international peace, rather it means such policies should protect people’s basic human rights too. The assumption behind economic sanctions is that economic pressure on the population of a country forces the government to reconsider its policies. The statement that economic sanctions do not target humanitarian goods seems incorrect when they aim to diminish the main source of a state’s revenue. The level of realization of human rights is dependent to the state’s income level. Therefore, economic sanctions endanger current level of people’s enjoyment of their right to health by decreasing available resources of a country to be spent on the health of population.

Despite the international community’s statement that sanctions are directed at the government of Iran for its nuclear program, during the sanctions period, the enjoyment of Iranians of their fundamental rights has been dramatically decreased. The sanctions affected health of Iranians in two ways; first by deteriorating their living conditions through rise of inflation and unemployment, and decline of households’ income and access to adequate nutritious food and healthcare; then, by direct effect on the availability, accessibility and quality of lifesaving medicines. Humanitarian exemptions which were established after the call of shortage could not protect the population from adverse effects of sanctions. If the purpose of sanctions has been refraining Iran from developing nuclear weapons, sanctions should have been all about the materials and technology related to this program and targeted decision-making elites, not ordinary people. Although, it seems that the world overlooked countries that actively have nuclear weapons without ratification of Non-Proliferation of Nuclear Weapons Treaty.

According to international human rights laws, right to health is a justiciable right.^134^ The countries imposing sanctions, UN treaty bodies and Iran itself are responsible for the humanitarian effects of sanctions on Iranians’ lives. Iran could not appropriately handle the humanitarian crisis caused by sanctions and still be able to maintain the level of its population’s enjoyment of their basic rights. The country should have predicted the impacts of sanctions and planned for the protection of its population. But some of the consequences of the sanctions such as the difficulty in access to medicines (which were in theory exempted from sanctions) were difficult to be predicted. It shows that laws on “humanitarian exemptions” solely do not protect the rights of the population of targeted countries. On the other hand, through joining UN and ratifying human rights treaties, almost all countries around the world are committed to respect human rights of everyone without any kinds of discrimination. No international human rights treaty questions the equality of human beings in their inherent dignity and fundamental rights. It is thus against all these treaties to assume that violation of people’s rights in one country, through pressure of sanctions is acceptable. Olivier De Schutter, UN Special Rapporteur of the right to food in his report of 2011 stated that a state who uses its means of influence, such as its economic leverage to induce policies in another state’s jurisdiction that undermines the targeted state’s human rights obligations, is responsible for violation of rights under international law.^135^ Iran has not been the first country that has faced sanctions; therefore the adverse impacts of sanctions on this country could be predictable by UN Security Council and by the countries which imposed them. In future, prior to the imposition of any economic sanctions, the international community must use effective measures to protect the human rights of target country’s population. Countries should also use every political and legal means to prevent violation of the rights of these people by other countries or international organizations. International laws related to economic sanctions also need improvement. All the UN organs’ resolutions on economic sanctions are similar in content and are not deterrent enough. These resolutions are soft laws advising targeting and targeted countries to respect human rights. However, no international accountability system is established for countries which impose economic sanctions and do not conform to these laws. According to General Comment no 14 ICESCR, effective judicial and appropriate remedies at national and international levels should be provided for people whose rights are violated.^20^ Even following dissemination of the report of the Special Rapporteur on Human Rights and UN General Secretary about humanitarian effects of sanctions on Iran, the imposing countries were not compelled to lift sanctions. The UN as a “center for harmonizing the actions of nations in the attainment of peace”^16^ should take a clear position about bilateral sanctions or “measures” such as these, imposed arbitrarily by countries or groups of countries against another one.

Moreover, in the case of Iran, humanitarian exemptions were decided too late, while the process of implementation of the exemptions was not clear. Prior to the imposition of sanctions, an international order for the protection of people should be established and some international intermediate organizations and certain companies and financial institutions should be designated to facilitate the implementation of exemptions. Furthermore, the effects of sanctions should be continuously monitored; if basic human rights are affected, the sanctions should be reviewed. Economic sanctions in their current method of implementation are “collective punishment” and violation of all human rights treaties. By putting such great pressure on the population of a country, other countries will not also be safe. The prevalence of diseases and internal conflicts in a country, as results of economic sanctions, are threats to international health and peace. People who find their rights violated seek asylums in other countries. Considering human rights approach in foreign policies might be a better solution than economic sanctions for resolving international disputes.

Recently, a proposal is suggested by imposing countries to lift sanctions on Iran due to a shift in Iran’s nuclear policies. However, the adverse consequences of economic sanctions have already taken place and will take a long time to be alleviated, if not impossible. The social impact of economic sanctions against Iran may extend beyond the sanction period because the costs of imposing sanctions exceed the benefits of lifting sanctions. Moreover, lifting sanctions necessarily will not lead to improving living standards and welfare of Iranians if this country does not invest more on the population. After improving access to medicine and allocating more funds to health system, now, Iran needs to make considerable efforts to improve the living conditions of the people, especially the poor and children by using all the necessary means and recourses available. In addition, this country must consider new policies to protect civilians from the violation of their rights in similar situations. Imposing countries and the international community are also responsible for the humanitarian consequences of their actions and should aid Iran by any appropriate or necessary means to alleviate the situation and to allow Iranians to enjoy their human rights which have been affected by the sanctions.

## Ethical issues

Not applicable.

## Competing interests

Author declares that she has no competing interests.

## Author’s contribution

FK is the single author of the paper.
